# Phenomenological Model for Defect Interactions in Irradiated Functional Materials

**DOI:** 10.1038/s41598-017-05071-z

**Published:** 2017-07-13

**Authors:** Steven J. Brewer, Cory D. Cress, Samuel C. Williams, Hanhan Zhou, Manuel Rivas, Ryan Q. Rudy, Ronald G. Polcawich, Evan R. Glaser, Jacob L. Jones, Nazanin Bassiri-Gharb

**Affiliations:** 1 G.W. Woodruff School of Mechanical Engineering, Georgia Institute of Technology, Atlanta, GA 30332 USA; 2 Electronics Science and Technology Division, US Naval Research Laboratory, Washington, D.C. 20375 USA; 3 School of Materials Science and Engineering, Georgia Institute of Technology, Atlanta, GA 30332 USA; 4 Department of Materials Science and Engineering, North Carolina State University, Raleigh, NC 27695 USA; 5 Sensors and Electron Devices Directorate US Army Research Laboratory, Adelphi, MD 20783 USA

## Abstract

The ability to tailor the performance of functional materials, such as semiconductors, via careful manipulation of defects has led to extraordinary advances in microelectronics. Functional metal oxides are no exception – protonic-defect-conducting oxides find use in solid oxide fuel cells (SOFCs) and oxygen-deficient high-temperature superconductors are poised for power transmission and magnetic imaging applications. Similarly, the advantageous functional responses in ferroelectric materials that make them attractive for use in microelectromechanical systems (MEMS), logic elements, and environmental energy harvesting, are derived from interactions of defects with other defects (such as domain walls) and with the lattice. Chemical doping has traditionally been employed to study the effects of defects in functional materials, but complications arising from compositional heterogeneity often make interpretation of results difficult. Alternatively, irradiation is a versatile means of evaluating defect interactions while avoiding the complexities of doping. Here, a generalized phenomenological model is developed to quantify defect interactions and compare material performance in functional oxides as a function of radiation dose. The model is demonstrated with historical data from literature on ferroelectrics, and expanded to functional materials for SOFCs, mixed ionic-electronic conductors (MIECs), He-ion implantation, and superconductors. Experimental data is used to study microstructural effects on defect interactions in ferroelectrics.

## Introduction

Modification of the type, charge, concentration, spatial distribution, and mobility of defects in a material can result in dramatic effects on functionality^1^. Semiconductor materials are a prime example, where strict control over defect type and density is used to modify electronic and optical properties^1, 2^, resulting in the design of devices that we use and interact with on a daily basis, from modern *pn*-junction transistors to photovoltaic (PV) cells and photoactive image sensors^2, 3^. Similar control in functional oxides is substantially more challenging to implement, where local charge balance and phase stability are intertwined with material functionality. For example, in high-temperature superconducting ceramics, localized material defects can interact with and pin magnetic vortices, thus enhancing superconducting properties^4^. Strong interactions between oxygen-related defects in proton-conducting oxides result in crucial changes to conduction behavior for mixed ionic-electronic conductors (MIECs) and solid oxide fuel cells (SOFCs)^5^. In ferroelectric materials, defect interactions, such as those between domain walls (2D defects) and point defects (0D) are often the major contributor to substantial dielectric, ferroelectric and piezoelectric responses. Various methods have attempted to elucidate such interactions, both theoretically and empirically^6, 7^. In this work we demonstrate a universal approach for quantifying defect-defect interactions in functional materials, and specifically ferroelectrics, without chemical doping, but rather achieved through total ionization dose (TID) studies. A phenomenological model is implemented for such quantification, with the ultimate goal of becoming a tool in defect engineering and functional response control of these oxides. The proposed model is successfully demonstrated for direct comparison of defect-defect interactions in a range of ferroelectric compositions in both thin film and bulk form. Further applicability is demonstrated by fitting the model to data on functional materials for applications in solid oxide fuel cells (SOFC), mixed ionic-electronic conductors (MIECs), He-ion implantation, and superconducting oxides. Subsequently, the model is employed to compare the effects of grain structure on the interaction of domain walls with defects in polycrystalline lead zirconate titanate (PZT) thin films.

## Background

Since the inception of the transistor, defect engineering of semiconductor materials has undergone momentous advancements, leading to a continuous revolution in electronic devices. The transformative nature of precise defect control and design in these materials has resulted in an abundant manipulation of the resulting material functionalities, which has in turn allowed for vast reductions of length scales for resulting devices. Metal oxide materials offer a substantially wider range of functionalities, with many of them coexisting in the same material. However, these materials suffer from a greater degree of complexity that has hindered similar levels of control over impurity-driven properties^1, 4^. Inclusion of more than one elemental component, chemical instability, and diffusion often result in complex interactions of defects with oxides’ functional properties^4, 8, 9^, making such material systems increasingly difficult to control and optimize, specifically in polycrystalline thin films^1^. Such challenging control of defects in functional oxides has often translated into the notion that defects are deleterious to material functionalities^10^. This is particularly relevant in ferroelectric oxides, where defects are often considered to be a source of degradation of dielectric, ferroelectric, and electromechanical responses^11^. Indeed, the elastic and electrical energies associated with defects can affect the motion of internal interfaces, such as hysteretic and nonlinearly-mobile domain walls and phase boundaries: i.e., the pinning energy for the motion of domain walls is defined by the local defects, resulting in elastic and electrical fields in the lattice^11–13^. The mobility of these internal interfaces is largely responsible for the very large functional responses exhibited by ferroelectric materials, and the restriction thereof by defect interactions results in considerable degradation of functional properties^11–14^. However, recent reports have demonstrated that the introduction of defects via ion bombardment in near-perfect single-crystal ferroelectrics can, in fact, *enhance* ferroelectric properties^15^. Likewise, defects in yttrium barium copper oxide (YBCO) can pin magnetic flux, resulting in increased supercurrent properties^4^. As such, it is imperative to further develop methods for investigating and manipulating structure-property relationships wrought by defect interactions in complex functional oxides.

Traditionally, the effects of defect concentrations in functional materials are studied via impurity doping, an approach historically used for manipulation of material functionalities^16, 17^. For example, in PZT, acceptor dopants (e.g. Fe^3+^, Mn^3+^) create oxygen vacancies and defect dipoles which can pin domain wall motion, degrading electromechanical response, while reducing dielectric, dynamic mechanical, and piezoelectric losses in the material^18^. Conversely, donor dopants (e.g. La^3+^, Nb^5+^) introduce excess positive charge, compensated by Pb vacancies, as well as reduced number of oxygen vacancies and defect dipoles in the material^18, 19^. Such reduction leads to increased domain wall motion and results in greater electromechanical response, but also larger dielectric losses^18, 19^.

A variety of work has been carried out to quantify interactions between defects and the resulting effects on the functional response of ferroelectrics. First and foremost, the Rayleigh analysis, originally introduced for ferromagnetic materials and adapted to ferroelectrics in late-1990s by Taylor and Damjanovic, is currently widely employed to quantify contributions of domain wall (2D defects) motion to dielectric responses in ferroelectric materials^7, 20^. However, while the Rayleigh analysis allows a quantification of the overall extrinsic contributions to the functional response, it does not directly quantify trends in functional response as a function of defect concentration. In the late 1980s, Kronmüller showed an approximately inverse proportionality between domain wall motion and defect concentration in ferromagnetic materials; Boser expanded this work to ferroelectric BaTiO_3_, demonstrating a similar inverse relationship between dielectric permittivity and dopant concentration^6, 21^. While the changes associated with dopant-induced defects can be substantial, it is often difficult to distinguish whether changes to material properties are the result of the complex effects of compositional heterogeneity, defect interactions, or a combination of both. Manipulation and study of defect interactions via irradiation provides a method that forgoes chemical doping and its associated complications, e.g., due to phase separation and instabilities arising from chemical heterogeneity in the material^22^. Radiation-induced ionization and displacement events can increase defect concentrations, causing changes in defect energy and potentially modifying functional responses of functional oxides^11, 13, 23^. The ability to quantify these radiation-induced defect concentrations and accurately predict material response as a function thereof has enormous implications not only for ferroelectric materials, but also for defect engineering of a variety of other functional metal oxides^24^, for applications ranging from multiferroic multistate memory devices and optoelectronics^25, 26^, to mixed ionic-electronic conductors^5, 27, 28^, superconductors^4, 29^, and even nuclear fusion materials and X-ray applications^30^.

## Phenomenological Model for Defect Interactions in Functional Oxides

Prior work on irradiation of functional materials has generally shown a direct correlation between radiation dose and degradation of functional properties^31–38^. Specifically, trapped charges and ionic displacements – activated or generated as a result of X-ray, gamma, electron, proton, neutron, and heavy-ion irradiation at intermediate and high doses – can result in degradation of functional responses through changes to defect concentration, defect interactions, and the defect energies in the material^15, 23, 35, 39–42^.

Here, we present a phenomenological model to quantify such defect interactions by assuming that defects, either generated or activated in the material by irradiation, will eventually result in measurable changes to the overall material response. We consider active defect interactions to encompass both an interaction between a created/activated defect and functional material volume, as well as interaction between defects that result in modifications of extrinsic contributions to the functional response (such as domain wall motion in ferroelectric materials, proton-conducting perovskites, etc.)^43^. Such interactions will inevitably impact a given region of the material, defined here as the volume *V*_*def*_. Conversely, a “free” material volume is defined, *V*_*free*_, corresponding to regions where defects are either not present or are present but do not result in measurable changes to the functional response. We further assume that the number of defects in an arbitrary volume of the sample, *N*, changes in proportion to radiation dose, thus allowing for correlation of degradation/enhancement of functional response to changes in *V*_*def*_ from an easily-quantifiable and manageable external “stimulus”. We can relate the change in *V*_*def*_ to changes in the number of defects, *N*, as a function of: (1) the volume fraction of the material where defect interactions are free to occur: *V*_*free*_/*V*_*T*_, where *V*_*T*_ is the total material volume; and (2) the mean change in material volume impacted per new defect created/activated, *V*_*N*_.

1 $$\frac{d{V}_{def}}{dN}={V}_{N}\frac{{V}_{free}}{{V}_{T}}$$

**Figure 1:**
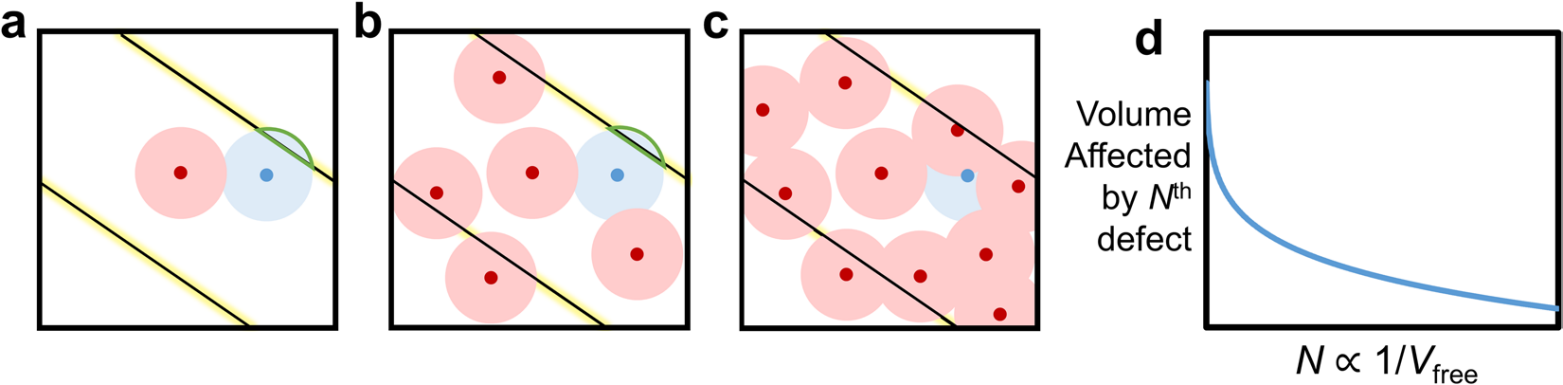
Schematic 2D simplification of 3D samples, illustrating the relationship between material volume, volume affected by defects, and the effects of new defects. (**a**) Shown is a single grain with three domains, separated by two domain walls. Free material volume is shown in white, pre-existing defects and the volume they affect in red, and a newly-introduced defect in blue. A larger free volume results in greater mean volume pinned by the new defect. (**b**,**c**) With increasing number of pre-existing defects (**d**) the volume pinned per new defect is reduced. Furthermore, the weighting function is employed to account for nonlinear defect interactions, such as those between defects and domain walls shown in green in (**a**) and (**b**). As available sites for new defects to impact domain walls are reduced with greater numbers of pre-existing defects, the weight of new-defect impact on free material volume is modified. The available domain wall area for interactions with new defects is highlighted in yellow.

Equation (1) expresses the fact that the volume of material impacted by new defects is proportional to the fraction of free volume in a given material – larger free volume yields a greater volume influenced by a new defect, due to more free “sites” (Fig. 1). However, the presence of internal interfaces and material nonlinearities, such as grain boundaries, pre-existing defects, chemical heterogeneity, etc., may result in deviations from the mean volume that a new defect will impact. Thus, we introduce a generalized weighting function, *W*(*N*) to account for material-specific fluctuations to the actual volume impacted by each new defect created/activated, *V*_*N*_, and the resulting modifications of extrinsic contributions to the functional response. The weighting function is also expected to depend on the number of defects created/activated, *N*, which is proportional to the radiation dose. Thus,

2 $$\frac{d{V}_{def}}{dN}={V}_{N}W(N)\frac{{V}_{free}}{{V}_{T}}$$

Further details on the above derivation are available in the Supplementary Information. Using the relationship *V*_*def*_ + *V*_*free*_ = *V*_*T*_, we can substitute for the free volume, *V*_*free*_, arriving at

3 $$\frac{d{V}_{def}}{dN}={V}_{N}W(N)\frac{{V}_{T}-{V}_{def}}{{V}_{T}}={V}_{N}W(N)(1-\frac{{V}_{def}}{{V}_{T}})$$

We can express the result in (3) as a normalized volume of material impacted by defect interactions, which we call *V*_*d*_:

4 $$\frac{{V}_{def}}{{V}_{T}}={V}_{d}$$

Appropriate substitution into both sides of Equation (3) and simplification yields:

5 $$\frac{d{V}_{d}}{dN}=\frac{{V}_{N}}{{V}_{T}}W(N)(1-{V}_{d})$$

The constant *V*_*N*_ is normalized per total volume *V*_*T*_, yielding φ_*N*_, the normalized mean change in material volume impacted by defect interactions per new defect created/activated. Separation of variables and integration results in:

6 $${V}_{d}=1-{e}^{-{\phi }_{N}\int W(N)dN}$$

Given the large dependence of the functional properties on the extrinsic contributions, we can expect the changes in functional response as a function of TID to be directly dependent on the material volume affected by defect-defect interactions. The resulting relationship is a generalized form that can be applied to a variety of functional materials that rely on active/inactive defects within the material volume to drive their functional properties^4, 5, 15^. We note that similar expressions have been previously derived for defect motion and interactions in various semiconductor and insulator materials, including Si, SiO_2_, and graphene^37, 38, 44^. Additionally, Equation (6) bears resemblance to the sigmoid function used to describe phase transition behavior in solids from the Johnson-Mehl-Avrami-Kolmogorov equation^45^.

To further develop expression (6), a knowledge of the weighting function is needed. The proposed expression that relates material nonlinearities to the magnitude of material volume impacted by a new defect (either created or activated by irradiation), thus resulting in modification of the functional response, is:

7 $$W(N)=\frac{1}{{N}^{k}}$$

where *k* is a fitting coefficient related to the rate of defect saturation in the material, which is discussed hereafter. We note that the proposed weighting function is consistent with prior work in both ferroelectric and ferromagnetic materials, for defect interactions between domain walls and chemically-induced (dopant) point defects. Kronmüller originally demonstrated an *N* ^−1^ relationship for degradation of domain wall motion in multiaxial ferromagnetic crystals as a function of density of point defects^21^. Boser expanded this work to ferroelectric BaTiO_3_, showing an inverse (*N* ^−1^) relationship of the irreversible Rayleigh parameter (a measure of domain wall mobility) on defect concentration as a result of Fe doping^6^.

Compared to the above work, we introduce an effective, fitted coefficient, *k*, in order to account for more complex material variations, which implies, for ferroelectric materials, domain size and orientation, grain boundary density and morphology, the effects of defect-influenced volumes on neighboring regions, number of defects necessary to modify response in a given volume, etc. Thus, *k* encompasses the variety of inherent material effects that will lead to deviations from an exact *N* ^−1^ relationship of new defects contributing to response-altering defect interactions in a given discrete ferroelectric material volume. Furthermore, while Boser and Kronmüller demonstrated *N* ^−1^ dependencies of domain wall motion on defect concentrations, thus implying only degradation of properties, previous work on chemical doping as well as recent radiation work by Saremi *et al*., has shown enhancements to functional properties of ferroelectric materials, suggesting that deviations from a perfectly inverse decay are possible^15, 46^. Thus, the use of the effective parameter *k* offers relative flexibility regarding material behavior as a function of the number of defects (and specifically, TID). Inserting the weighting function into (6), and solving, we arrive at a functional form:

8 $${V}_{d,func}=1-{e}^{-{\phi }_{N}(\frac{{N}^{1-k}}{1-k})}$$

For example, using the above expression, a virgin ferroelectric sample with no defects introduced by radiation (*N* = 0) yields *V*_*d*,*func*_ = 0, meaning that the functional material volume affected by newly-created defect interactions is zero in the pristine, virgin state. This model can be used to study the degradation trend data as a function of radiation dose or other external “stimulus” (dopant concentration, for example) that changes the number of defects per arbitrary volume (concentration, assumed proportional to external “stimulus”). We observe that while newly-created or -activated defects are assumed to interact with a constant volume, φ_*N*_, this parameter does not account for factors such as proximity to pre-existing defects or material anisotropy, etc. The effective rate of saturation, *k*, is able to account for these deviations from linear degradation of response. This effect is typically observed as an asymptotic trend in the response, where subsequently-formed defects result in reduced contributions to the overall change in response.

To correlate the above expression with the functional response of the material, we again consider the specific case of ferroelectric materials. The major contributor to the reduced functional response in irradiated ferroelectric materials has been shown to be degradation of the extrinsic response, i.e. increase in defect interactions with ferroelectric material volume through increased pinning of the domain walls by radiation-induced defects^35^. We note that active defect interactions resulting in measurable changes to material response do not necessarily pin all functional response, but “pinned volume” is used hereafter to refer to volumes of the material in which defect interactions produce quantifiable changes in functional response.

**Figure 2:**
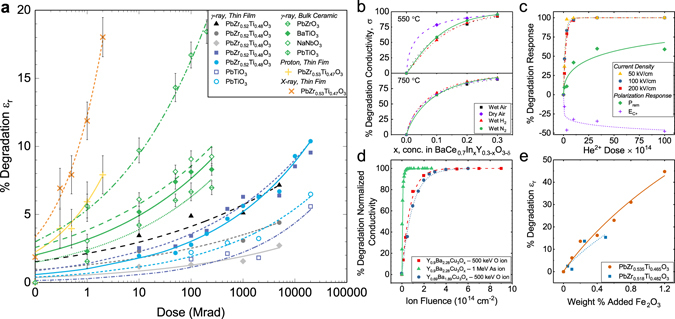
Application of the proposed model to previously-reported total ionizing dose (TID) studies in ferroelectrics and to dopant/radiation studies on various functional materials. (**a**) The effects of gamma, X-ray, and proton irradiation on the dielectric permittivity of various bulk and thin film ferroelectrics are shown. Data in gray/black shows gamma dose rate studies^47^. Blue symbols indicate degradation of the dielectric permittivity at positive and negative coercive voltages for PZT and lead titanate^48^. Green symbols show experiments on bulk ferroelectrics performed^49^. X-ray and proton irradiation of PZT shown in orange and yellow symbols, respectively^39^. (**b**) Indium doping in BaCe_0.7_In_x_Y_0.3−x_O_3−δ_ at different atmospheric conditions^28^. (**c**) He-ion implantation in epitaxial perovskite PbTiO_3_ films^15^. (**d**) O- and As-ion irradiation of superconducting yttrium barium copper oxide (YBCO)^23^. (**e**) Fe_2_O_3_ doping of ferroelectric lead zirconate titanate (PZT)^50^. All data reproduced with permission.

If exposure to radiation results in defect creation, which translates into degradation of the functional properties, such degradation can be measured as a function of TID (where degradation is plotted as a positive value) (Fig. 2). Hence, φ_*N*_ is positive for a degradation trend, and thus results in positive values of pinned volume, *V*_*d*,*func*_, when substituted into Equation (8). Conversely, a negative value for φ_*N*_ represents an enhancement of response (due to an effective “reduction” of pinned volume). A flat degradation curve would correspond to φ_*N*_ = 0, indicating no decay in properties due to irradiation. A larger magnitude of φ_*N*_ signifies a greater interaction of the radiation-induced defects with the material, and increased degradation/enhancement of the functional properties. Therefore, φ_*N*_ can generally be considered as the global susceptibility of the material to radiation-induced defects.

We further note that both the φ_*N*_ and *k* parameters can be highly dependent on the specific microstructure, grain morphology, existing defects and charge, etc. In order to demonstrate the viability of the proposed model and weighting function to study radiation-induced defect interactions and defect engineering of functional oxides, we apply the model to prior literature reports on the effects of various types of radiation on multiple functional materials. Figure 2a shows the applicability of the proposed model to degradation of the dielectric permittivity in a range of ferroelectric materials exposed to irradiation under various measurement conditions^39, 47–49^. The φ_*N*_ and *k* fitting parameters for the data are reported in detail in Supplementary Tables 1 to 6. We note that the model is therefore applicable to a variety of radiation types – both ionizing (e.g. gamma, X-ray) and heavy particles (e.g. protons, neutrons) – as well as multiple functional properties, including dielectric, polarization, and electromechanical responses, as demonstrated in Supplementary Figures 2 to 7. The model is further expanded to include a broader class of defects in functional materials, demonstrating extensive applicability to doped barium cerate proton conductors for use in solid oxide fuel cells (SOFCs) (Fig. 2b)^28^, He^2+^-ion implantation in epitaxial perovskite lead titanate (Fig. 2c)^15^, and heavy ion irradiation/implantation of yttrium barium copper oxide (YBCO) high-temperature superconductors (Fig. 2d)^23^. The model is also applicable for defect generation via chemical doping, as shown for examples of various impurity-doped ferroelectric ceramics (Fig. 2e and Supplementary Figures 8 and 9 and Supplementary Tables 7 and 8)^50, 51^ and X-ray synchrotron characterization of functional metal oxide memory cells (Supplementary Figure 13)^42^.

We note that the use of this phenomenological model allows for a quantitative comparison of the effects of various irradiation experiments on the materials’ properties. For example, as evident by the qualitative data trends, the large degradation in the properties of PZT thin films exposed to X-ray and proton radiation translates into greater effective pinning of ferroelectric material volume, φ_*N*_, compared to those exposed to gamma irradiation (Fig. 2 and Supplementary Tables 1 to 6). The result is a potentially greater number of radiation-induced defects that lead to increased pinning of material volume in X-ray-irradiated samples (compared to gamma), reflected by the greater material volume affected by defect-defect interactions, φ_*N*_ (Supplementary Table 5). Further discussion of linear energy transfer (LET) of various radiation types and its relation to defect generation/activation is available in the Supplementary Information.

It is also interesting to note that the bulk ferroelectric samples’ dielectric permittivity is more susceptible to gamma radiation exposure than thin film ferroelectric samples. We note that both end members of lead zirconate titanate solid solution are studied in the bulk form, and therefore the effects of Zr:Ti ratio (relative to other ferroelectric compositions) might be minimal. However, in general, the bulk ceramics show a larger value of φ_*N*_ (Supplementary Table 4) than the thin films exposed to gamma-rays. Additionally, the values of *k* for gamma-irradiated bulk samples are among the highest shown in Fig. 2a, with the exception of the work from Zhang *et al*.^47^. The average grain size in polycrystalline thin films is typically smaller than in bulk ceramics (50–150 nm in thin films, compared to micron-sized grains in bulk ceramics), thereby increasing both grain and domain density in thin films^52^. The extent of pinning of newly-created/activated defect centers in samples with smaller grains/domains is potentially more contained than in samples with larger grains, i.e. smaller morphological features enact physical constraints on growth of defect-pinned regions. In other words, in bulk ferroelectrics with larger areas of (nearly) homogenous crystallinity, newly-formed defects may result in greater interactions and longer range effects on domain wall motion (Fig. 1b and c). Thus, in bulk samples with larger grains, not only should the volume pinned per new defect be greater, but saturation of response degradation should occur more quickly, as new defects are free to affect a larger volume without “containment” by grain boundaries.

Additionally, Daniels *et al*. have previously shown minimization of electrostatic potentials at grain boundaries in ferroelectric materials due to interactions of domains at the same locations^53^. In irradiated ferroelectrics, such minimization could potentially reduce the deleterious effects on functional properties caused by radiation-generated charged defects. In polycrystalline samples with smaller grains and higher grain boundary density, this effect is enhanced relative to bulk samples with larger grains, and trends of milder degradation are observed. Namely, the mean volume pinned per new defect can be reduced, as charged defects are annihilated or compensated at the grain boundaries.

The above data support the fact that microstructural features play a critical role in defect interactions in ferroelectrics. To further elucidate this observation, in the following section we compare the effects of gamma radiation on polycrystalline PZT films with columnar and equiaxed grains.

## Effect of Microstructure on Defect Interactions in Ferroelectric Thin Films

**Figure 3:**
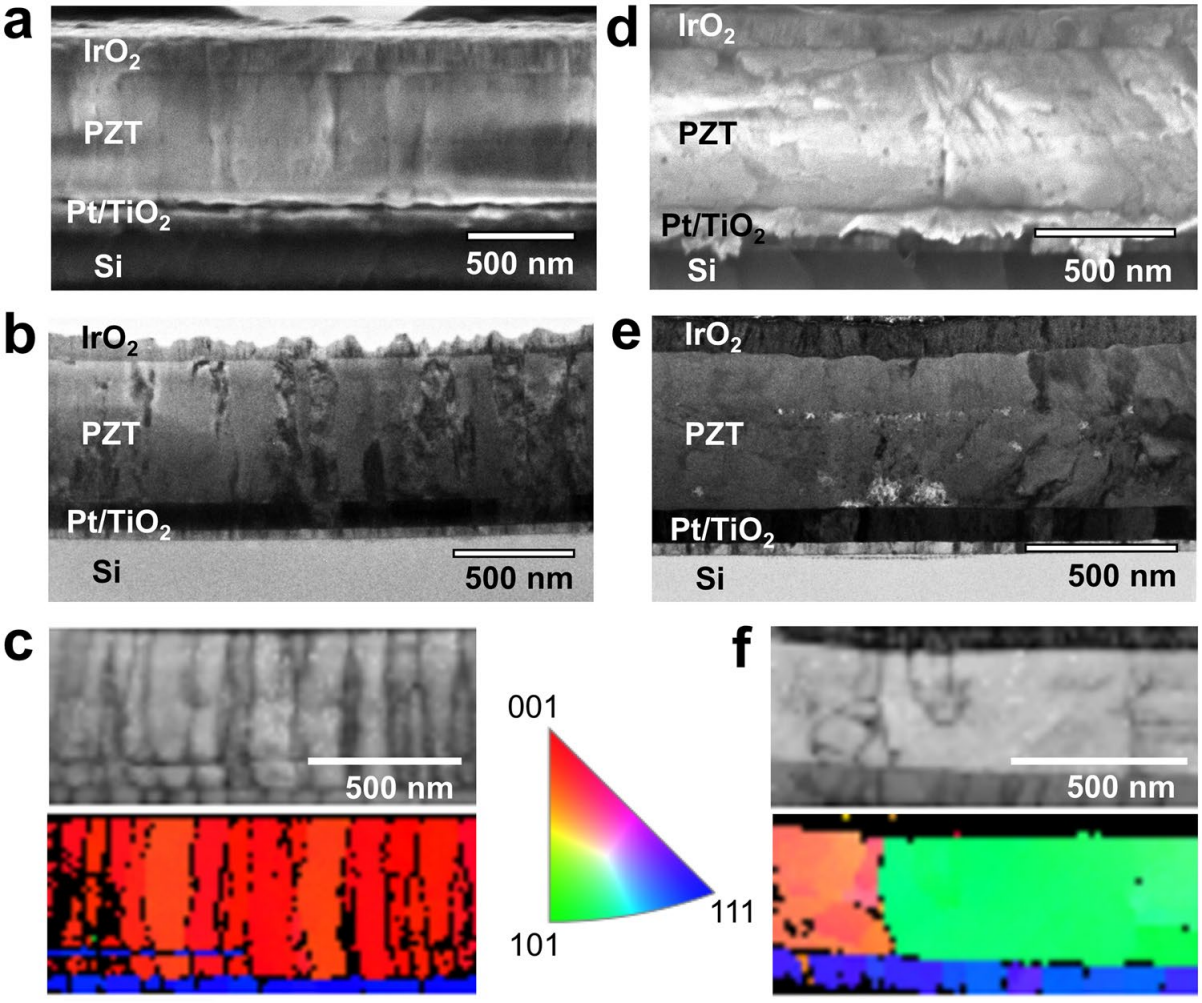
Microstructural characterization of PZT thin films with columnar and equiaxed grains. (**a**,**d**) SEM, (**b**,**e**) TEM, and (**c**,**f**) TKD cross-sectional images of columnar-grained films (**a**,**b**,**c**) processed using 2-methoxyethanol-based precursor PZT solution, and equiaxed-grained films (**d**,**e**,**f**) prepared using a methanol-based inverted mixing order PZT precursor solution. Note that the crystallographic poles noted for the pole figure are relative to the film normal (verticle direction in the image).

**Figure 4:**
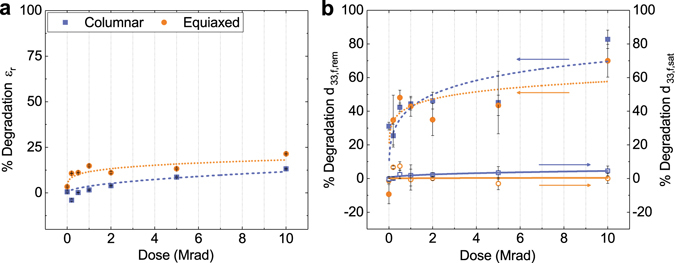
Application of the proposed model (curves) to experimental data from PZT thin films with columnar and equiaxed grain structure. (**a**) Plots of relative, low-field dielectric permittivity, and (**b**) saturation and remanent piezoelectric as a function of radiation dose^62^, and the fitted model to each data set. Error bars show standard error of the sample mean. Fitted parameters are detailed in Table 1.

PZT thin films with columnar and equiaxed grain structures were fabricated via chemical solution deposition (CSD) and exposed to ^60^Co gamma radiation, from 0.2 to 10 Mrad, at a dose rate of approximately 600 rad(Si) s^−1^. Measurements to probe the low-field relative dielectric permittivity (ε_r_) as well as the remanent and saturated converse, effective, longitudinal piezoelectric responses (*d*_*33*,*f*,*remanent*_ and *d*_*33*,*f*,*saturation*_, respectively) were performed before and, at discrete dose intervals, after irradiation. Scanning electron microscopy (SEM), transmission electron microscopy (TEM), and transmission Kikuchi diffraction (TKD) were carried out (Fig. 3) to observe and quantify grain orientation and size statistically (Supplementary Table 12). Interpretation of the TKD results indicates primarily 100-texture in samples with columnar grains (Fig. 3c), while the samples with equiaxed grains are more randomly oriented, i.e. weaker texture (Fig. 3f). The degradation trends for dielectric permittivity, *ε*_*r*_, and effective longitudinal piezoelectric coefficient, *d*_*33*,*f*_, as a function of TID, as well as the corresponding phenomenological models for the defect-defect interactions they represent are shown in Fig. 4. The corresponding fitting parameters φ_*N*_ and *k* are reported in Table 1; full data sets are available in Supplementary Tables 13 and 14.

**Table 1:** Extracted parameters from application of phenomenological model to TID studies.

	Columnar		Equiaxed	
	φ_*N*_ × 10^3^	*k*	φ_*N*_ × 10^3^	*k*
ε_r_	19	0.48	26	0.79
d_33,f, remanent_	185	0.66	142	0.77
d_33,f, saturation_	7	0.60	1	0.62

Shown are dielectric permittivity and effective longitudinal piezoelectric response in gamma-irradiated PZT thin films. Data is extracted by fitting Equation (8) to percent change of given functional response measurement vs. radiation dose. Note that values of φ_*N*_ are multiplied by three orders of magnitude to make interpretation more manageable.

A comparison of the TID trend quantification for dielectric and electromechanical responses shows that the values for the change in volume pinned per defect, φ_*N*_, are comparable (same order of magnitude) for individual measurements on samples with columnar and equiaxed grains. However, focusing on response-specific trends, large effective volume pinned is observed for measurements of ε_*r*_ and *d*_*33*,*f*,*remanent*_ in both samples, while φ_*N*_ of *d*_*33*,*f*,*saturation*_ is somewhat negligible comparatively, for both samples. We note that the saturation values are measured under high DC electric fields, while the remanent values and the dielectric permittivity are measured under zero DC bias and low AC electric fields. The observed trend in φ_*N*_, therefore, suggests that defects activated via gamma irradiation are likely of relatively low energy. That is, at the low applied electric field used to measure remanent response, the pinning strength of the defects is sufficient to result in large degradation of functional properties. At the elevated applied electric field used to measure saturated electromechanical response (250 kV cm^−1^ DC bias), the drive for domain wall motion exceeds that of the defect, and the degradation of functional response is, at least partially, overcome, even at higher TID levels. Indeed, the values of φ_*N*_ for *d*_*33*,*f*,*saturation*_ measurements are very similar to those extracted from work by Gao *et al*., for measurements of dielectric permittivity of irradiated PZT thin films with an applied 133 kV cm^−1^ DC bias^54^. The implication that applied electric field potentially negates the deleterious effects of defects created by gamma irradiation is not new. However, the ability to quantify and compare high- and low-field degradation behavior of ferroelectrics with distinct morphologies across a range of radiation doses is particularly advantageous for tailoring material response to meet a variety of distinct operational requirements.

Interestingly, the effective rate of defect saturation, *k*, is consistently greater for samples with equiaxed grains than those with columnar grains. Claeys and Simoen have previously suggested that defects created or activated in functional materials by exposure to ionizing radiation are proportional to the surface area of grain boundaries with which they interact^55, 56^. A variety of reports on irradiation of functional oxide materials have shown increased radiation hardness for materials with increasingly small grain structures, and thus, higher grain boundary density^57, 58^. The samples with columnar grains in this work have a smaller volume than the samples with equiaxed grains, and a surface-area-to-volume ratio that is more than twice as large (Supplementary Table 12). Increased grain boundary density yields a greater number of sites for defect accumulation, acting as effective defect “sinks,” and potentially reducing their deleterious effects on functional response. The result is a slower rate of saturation of response degradation, as the effects of a portion of newly-formed defects are neutralized by the grain boundaries. Furthermore, the smaller grains and greater surface-area-to-volume ratio for columnar-grained samples may translate to better “compartmentalization” of functional material volume, thus limiting the effects of newly-created defects to smaller ferroelectric material volumes, and reducing the rate of defect saturation. These effects are both emphasized for samples with columnar grains, where *k* is observed to be closer to zero (linear decay), and is especially apparent in the decay of dielectric permittivity (*k* = 0.48) (Fig. 4a). Also noteworthy is the fact that the mean value of *k* for equiaxed samples (*k* = 0.73 ± 0.09) is in close agreement with the work by Kronmüller and Boser, as well as prior work published on irradiation of ferroelectrics, as discussed previously^6, 21^.

## Conclusions

In summary, we have demonstrated a phenomenological model for quantification of the effects of defect interactions in functional materials. While we have specifically demonstrated applicability to ferroelectric thin films to study the effects of microstructure on defect interactions with the material, the model presented is adaptable to a multitude of functional materials, i.e., wherever the functional response is derived from and/or is affected by some degree of defect interactions. We have further displayed the proposed model’s versatility by comparing data from the literature not only on bulk and thin film ferroelectrics, but also on a variety of functional oxides, including doped barium cerate for SOFCs, He^2+^-ion implanted epitaxial perovskite lead titanate, and heavy ion-bombarded superconducting YBCO. Furthermore, we show that irradiation of materials can be a strong alternative approach to chemical doping, due to the relative simplicity and favorable additive nature of radiation dose experiments. Correlation of materials’ properties to radiation dose allows for careful, on-demand modifications to properties and the ability to tailor functional response to the intended application. Ideally, the demonstrated method for quantification of defect interactions will stimulate further advances in defect engineering of functional materials. Studies on various grain morphologies, preferred crystallographic orientation, radiation types, measurement conditions, etc. will offer further application and refinement of the model proposed here. However, more important are the implications of radiation studies for direct evaluation and quantification of defect-defect interactions in functional materials without the complicated effects of chemical doping. The resulting theoretical and empirical knowledge regarding defect interactions encourages methods for defect engineering of a broad class of functional metal oxide materials.

## Methods

### PZT Thin Film Preparation

PbZr_0.52_Ti_0.48_O_3_ (PZT) thin films were fabricated at the US Army Research Laboratory using 150 mm-diameter 100-silicon wafers consisting of 100 nm Pt/35 nm TiO_2_/500 nm SiO_2_/Si^59^. A seed layer of PbTiO_3_ was deposited to induce 001-texture of the PZT thin films^60^. Two separate PZT sol-gel solutions were prepared, one via a 2-methoxyethanol (2-MOE) route at the US Army Research Laboratory, and the other using a methanol-based inverted mixing order (IMO) process at Sandia National Laboratories^60, 61^. Both 2-MOE-based and IMO solutions were deposited via chemical solution deposition, resulting in films with thicknesses of 500 ± 14 nm. IrO_2_ top electrodes were selected for continuity with prior work^35^; 100 nm thick electrodes were sputter-deposited onto the wafer at 500 °C and processed with a post-deposition anneal at 650 °C in flowing O_2_ for 30 min. The top electrode and PZT layer were patterned using argon ion milling and a series of additional metallization steps to create interconnects to device structures. This general process is outlined elsewhere^40^.

### Irradiation

The fabricated samples were exposed to radiation from a ^60^Co gamma source at doses ranging from 0.2 to 10 Mrad (equivalent Si dose) at a dose rate of approximately 600 rad(Si) s^−1^ at the Naval Research Laboratory. All electrodes were left floating during radiation exposure.

### Functional Response Characterization

Dielectric, polarization, and electromechanical responses of the samples were fully characterized at Georgia Institute of Technology both before and after irradiation, including measurements of low-field permittivity and DC electric field-dependent piezoelectric response, followed by irradiation and repetition of all experiments. A detailed summary of these measurements for samples with both columnar and equiaxed grain structures as a function of radiation dose is shown in Supplementary Tables 10 and 11, Supporting Information. A 600 second poling step at 10 V, approximately five times the coercive voltage, *V*_*c*_, was performed directly before the electromechanical measurements in both pre- and post-irradiation measurement sets in order to eliminate anisotropic polarization contributions to electromechanical response. All measurements were performed on the same sample/electrode both before and after irradiation in order to monitor precise changes in response behavior. Low-field dielectric permittivity (ε_r_) measurements were conducted at 100 mV and 1 kHz using an Agilent 4284 A precision LCR meter. Measurements of the converse, effective longitudinal piezoelectric response (*d*_*33*,*f*_) were performed on an aixACCT double beam laser interferometer (DBLI) measurement system up to 300 kV cm^−1^ DC bias with an overlapping AC signal *V*_*AC*_ ≈ 0.5*V*_*c*_. All measurements reported are subject to experimental error up to 3–5%, due to sample variability.

### SEM/TEM/TKD Analysis

Cross-sectional scanning electron microscopy (SEM), transmission electron microscopy (TEM), and transmission Kikuchi diffraction (TKD) (Fig. 3) were performed at North Carolina State University (NC State). Samples were sputter coated with a 20 nm-thick Au layer to increase sample conductivity during SEM imaging. Cross-sectional characterization was performed using an FEI Verios field-emission scanning electron microscope.

Before milling the samples under focused ion beam (FIB) for TEM and TKD, they were sputter coated with a 20 nm-thick Au layer to increase sample conductivity and to avoid drift. A 3 μm-thick Pt layer was deposited under FEI Quanta 3D FEG to protect the sample surface from electron and ion damage. Cross-sectional TKD samples were prepared under FEI Quanta 3D FEG using both electron and ion beam guns (SEM/FIB). TEM imaging was performed on the FIB samples using a JEOL 2000-FX scanning transmission electron microscope. Using an Oxford Instruments NordlysNano electron backscatter diffraction (EBSD) detector, Kikuchi diffraction patterns were mapped out under 30 kV with Oxford Instruments Aztec software. TKD data was processed using the HKL CHANNEL5 program Tango.

## Electronic supplementary material

Phenomenological Model for Defect Interactions in Irradiated Functional Materials
