# Supplementary material for: Phenomenological Model for Defect Interactions in Irradiated Functional Materials
Source: Sci Rep. 2017 Jul 13;7:5308. doi: 10.1038/s41598-017-05071-z (PMC5509665; doi:10.1038/s41598-017-05071-z)
Supplement: Supplementary file 1 — Phenomenological Model for Defect Interactions in Irradiated Functional Materials [file 41598_2017_5071_MOESM1_ESM.pdf]

Supplementary Information

Phenomenological Model for Defect Interactions in Irradiated Functional Materials

Steven J. Brewer[1], *Cory D. Cress*[2], *Samuel C. Williams*[3], *Hanhan Zhou*[4], *Manuel Rivas*[5], *Ryan Q. Rudy*[5], *Ronald G. Polcawich*[5], *Evan R. Glaser*[2], *Jacob L. Jones*[4], **Nazanin Bassiri-Gharb*[1,3]

[1] G.W. Woodruff School of Mechanical Engineering
Georgia Institute of Technology
Atlanta, GA 30332, USA
*Email: nazanin.bassirigharb@me.gatech.edu

[2] Electronics Science and Technology Division
US Naval Research Laboratory
Washington, D.C. 20375, USA

[3] School of Materials Science and Engineering
Georgia Institute of Technology
Atlanta, GA 30332, USA

[4] Department of Materials Science and Engineering
North Carolina State University
Raleigh, NC 27695, USA

[5] Sensors and Electron Devices Directorate
US Army Research Laboratory
Adelphi, MD 20783, USA

I. Radiation and Phenomenological Model

Generally, radiation interacts with a material by causing either ionization or displacement events. Lower-energy sources – e.g., X-rays and gamma rays – transfer energy to a material via electron interactions, ionizing the material with which they interact to form electron-hole pairs, and at higher energies, vacancy-interstitial pairs.[1,2] Irradiation with massive particles, such as protons and neutrons, transfers energy to a material through both electron interactions, as described previously, and atomic interactions, potentially resulting in displacement of atoms (vacancy-interstitial pairs) and subsequent defect cascades.[3]

In a ferroelectric material, radiation-induced ionization and displacement events can potentially increase the stable defect concentrations, including the trapping of charges at preexisting defects, thus modifying the energy associated with such defects' energy, their mobility, and other forms of interaction with the material. Prior work on the effects of radiation on ferroelectric materials has shown a direct correlation between total radiation dose and degradation of functional properties.[4-11] Specifically, trapped charges generated as a result of X-ray, proton, gamma and neutron irradiation can result in degradation of polarization, dielectric, and electromechanical response, primarily through changes to the energies of defects of the ferroelectric material.[11-15] In addition to ionizing energy transfer to the material, high-energy electrons and massive energetic particles (protons, neutrons, alpha-particles, etc.) also transfer energy via nuclear interactions that lead to direct atomic displacement. These radiation-induced displacements may degrade the functional ferroelectric response in a fashion that is qualitatively similar to that of ionization-related defects (especially for point defects like Frenkel pairs) or may give rise to unique degradation modes in the case of multi-atom defects (defect clusters) that span over larger volumes. Ultimately, radiation-induced modification of functional response in ferroelectrics is the result of changes in defect concentration and energy in the material. Therefore, irradiation can be leveraged as a method for controlled introduction and/or activation of defects in these material, thus modifying material properties and functional response, to meet the needs of a variety of applications. Such methods stand to benefit from robust quantification and phenomenological modeling to more adequately and conveniently compare material performance as a function of radiation.

Here, we offer such a model, beginning with the relationship describing the change in material volume affected by defect interactions, V_{def}, with the number of defects, N (assumed proportional to an external stimulus, such as radiation dose), which is a function of: (1) the volume fraction of the material that is *not* impacted by defects, V_{free}/V_T, where V_T is the total material volume; and (2) the mean change in material volume impacted per new defect created/activated, V_N;

$$\frac{dV_{def}}{dN} = V_N \frac{V_{free}}{V_T} \quad \text{(S1)}$$

When the volume fraction of free material is large, the effective volume impacted per new defect is maximized, due to the fact that there is greater free volume vulnerable to defects and overlap with pre-existing defects is less probable. Conversely, as the volume fraction of free material becomes small (i.e., reduced availability of free volume "sites" to be impacted), the probability of interaction and overlap with pre-existing defects is increased, and thus, the effective volume influenced by a new defect decreases (Figure 1). To account for material nonlinearities, such as grain boundaries, domain walls, pre-existing defects, etc., we introduce a weighting function, *W(N)*. The weighting function modifies the relationship between a newly-

introduced defect and the deviation from the mean volume it will impact, and thus contribute to increased defect interactions. Thus,

$$\frac{dV_{def}}{dN} = V_N W(N) \frac{V_{free}}{V_T} \tag{S2}$$

where V_N is a the mean volume pinned per defect introduced into the material and $W(N)$ is the weighting function. Using the relationship

$$V_{def} + V_{free} = V_T \tag{S3}$$

we can substitute for the volume of free material, arriving at

$$\frac{dV_{def}}{dN} = V_N W(N) \frac{V_T - V_{def}}{V_T} = V_N W(N) \left(1 - \frac{V_{def}}{V_T}\right) \tag{S4}$$

This expression is difficult to solve analytically without further simplification. We thus convert the expression for volume fraction to a normalized volume, thus eliminating the variable V_T and constraining the normalized volume impacted by active defect interactions, V_d to the range from 0 to 1:

$$\begin{gathered} \frac{V_{def}}{V_T} = V_d \\ V_d \in [0,1] \end{gathered} \tag{S5}$$

Substituting this result into expression (S4) requires multiplying the left-most part by V_T/V_T in order to convert the differential to the appropriate relation of dV_d:

$$\frac{V_T dV_d}{dN} = V_N W(N)(1 - V_d) \tag{S6}$$

Dividing V_T to the right-hand side of (S6) allows for normalization of the mean change in material volume impacted by defect interactions per new defect created/activated. We divide V_N by V_T to get φ_N. The result is

$$\frac{dV_d}{dN} = \varphi_N W(N)(1 - V_d) \tag{S7}$$

Separation of variables and integration results in

$$V_d = 1 - e^{-\varphi_N \int W(N) dN} \tag{S8}$$

Fitting the exponential function to degradation trends requires use of functional response data. Changes in functional response data are directly dependent on change of the volume impacted by defect interactions in the sample. Furthermore, testing the boundary condition, a virgin sample with zero defects (N = 0) yields a defective volume of zero. Further evaluation of expression (S8) requires integration of the weighting function (see full article). We use the weighting function

$$W(N) = \frac{1}{N^k} \tag{S9}$$

where k is a fitting coefficient related to the rate of defect saturation in the material. Solving, we arrive at

$$V_{d,func} = 1 - e^{-\varphi_N\left(\frac{N^{1-k}}{1-k}\right)} \tag{S10}$$

We can then use this to fit the degradation trend data and extract the φ_N and k parameters. Supplementary Figure 1 shows the effects of arbitrary changes to the φ_N and k parameters as a function of a given radiation dose. It is worth noting that fittings are done to the decimal value of degradation in response, e.g. for 5% degradation, a value of 0.05 is used for the fitting. Additionally, degradation is assumed to be positive, as it is the result of increases to the volume impacted by defect interactions. The result in Equation (S10) is robust and capable of fitting both degradation and enhancement data. This derivation assumes that the degradation trend data begins at zero change in response for zero defects/radiation dose. Realistically, materials contain inherent defects prior to irradiation. However, given the nature of percent changes in measured response of control samples, degradation trend data should conceivably show minimal change at zero exposure to radiation.

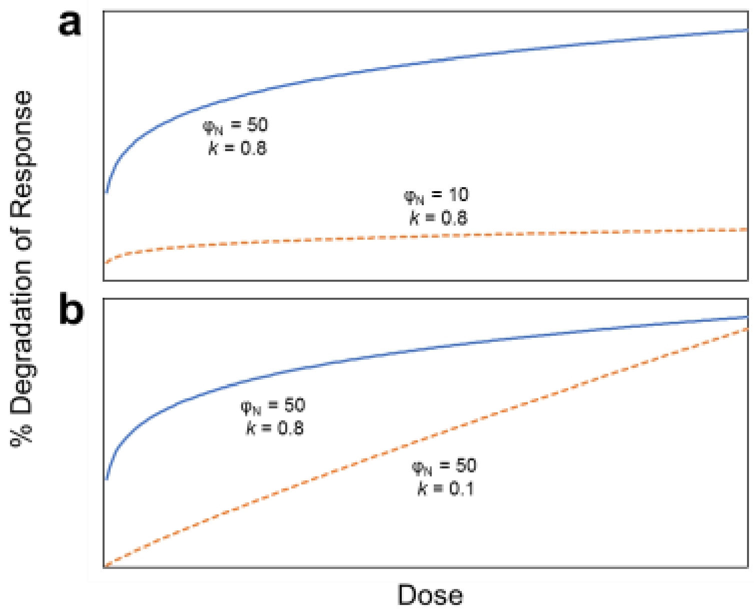

Supplementary Figure 1. Representative plots showing the effects of changes to the effective volume impacted by defect interactions, φ_N, and the effective rate of defect saturation, k, for degradation of an arbitrary response parameter. (a) shows changes to φ_N while holding k constant, and (b) shows the effects of changing k, while holding φ_N constant.

II. Application of Phenomenological Model to Literature Data

We apply the phenomenological model developed in this work to data reported in the literature from X-ray, gamma, proton, and neutron irradiation studies of various ferroelectric materials, both in thin films and bulk forms.[6,12,14,16-19] The results of fitting the model are tabulated in

Supplementary Tables 1 to 6, and results from individual studies are also shown in Supplementary Figures 2 to 7. Notably, by comparing the values of φ_N within individual publication data from the literature, the phenomenological model is able to consistently and accurately reflect the conclusions of the authors when studying a variety of processing and measurement conditions, including dose rate, bias conditions, and different radiation types. We have also applied the model to the results of chemical doping in various ferroelectric materials (Supplementary Tables 7 and 8, Supplementary Figures 8 and 9),[20,21] as well as ion irradiation of yttrium barium copper oxide (YBCO) superconductors (Supplementary Table 9, Supplementary Figure 10), He^{2+}-ion implantation of epitaxial ferroelectric thin films (Supplementary Table 10, Supplementary Figure 11), and In-doping of yttrium barium cerate proton conductors for solid oxide fuel cells (SOFC) (Supplementary Table 11, Supplementary Figure 12). Error bars are reproduced when available.

Studying the effects of various radiation types on ferroelectric thin films from Supplementary Figure 6 and Supplementary Table 5, we note that linear energy transfer (LET) in PZT for X-rays (10 keV), protons (3 MeV), and gamma rays (1.25 MeV) are 0.77 keV μm^{-1}, 38.25 keV μm^{-1}, and 0.05 keV μm^{-1}, respectively.[22-24] Notably, the LET for gamma radiation in PZT is much lower than both X-rays and protons. Comparing X-ray and gamma irradiation, the number of incident photons will be greater for gamma rays, but with a greater mean distance between them. Thus, fewer X-rays may impact the sample, but of those that do, more electron-hole pairs are generated. Additionally, at the lower energy of X-rays, there are many photoelectric lines (e.g., K, L, M, etc.) that potentially result in diverse ionization states of affected atoms, compared to simply ejecting outer shell electrons in the case of gamma irradiation.

On the other hand, the LET of protons is two orders of magnitude greater than that of X-rays. A greater dose rate conceivably translates to greater charge generation per unit volume, which could potentially increase the rate of recombination. Evidence of this effect is present in observed local enhancement of functional properties measured by Bastani et al., in samples irradiated with protons (Supplementary Figure 6).[12] Furthermore, smaller values of φ_N, the effective volume affected by radiation-induced defect interactions, in samples irradiated with protons compared to X-rays, support this hypothesis. Defects that could potentially degrade functional response are annihilated or their charge reduced to less-deleterious states, and the volume they pin is reduced. Additionally, the dose rate of protons is approximately 300 times that of X-rays, meaning 300 times fewer protons impact the surface than X-rays per unit time. Data from Oldham and McLean on irradiated MOS oxides demonstrated that the fractional hole yield for ionizing radiation (gamma, X-ray, electrons) was much greater than that of proton and alpha particle radiation, suggesting a more exaggerated interaction of ionizing radiation with the exposed material compared to particles.[25] The net result is a smaller mean volume affected by radiation-induced defect interactions in samples irradiated with protons compared to X-rays or gamma rays, where the fractional charge yield is greater (than that of proton irradiation) (Supplementary Table 5).

Supplementary Table 1. Extracted φ_N and *k* parameters from fitting equation (S10) to degradation data of various parameters from Zhang et al.[16] Note that values of φ_N are multiplied by three orders of magnitude to make interpretation more manageable.

**Zhang et al.**	**0.5 kGy hr^{-1}**		**2.5 kGy hr^{-1}**		**25 kGy hr^{-1}**	
	$\varphi_N \times 10^3$	*k*	$\varphi_N \times 10^3$	*k*	$\varphi_N \times 10^3$	*k*
ε_+	3	0.85	2	0.85	1	0.82

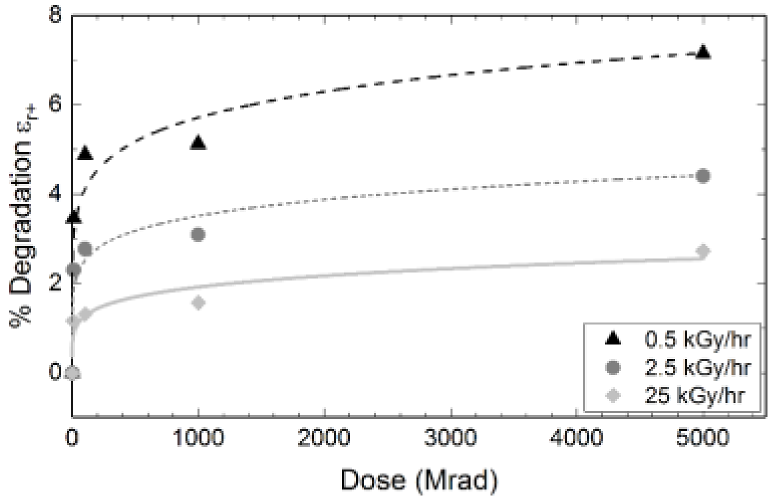

Supplementary Figure 2. Application of phenomenological model (curves) to work on the effects of gamma irradiation dose rate on degradation of dielectric permittivity measured at the positive coercive field (ε_{r+}) of PZT thin films.[16]

Supplementary Table 2. Extracted φ_N and k parameters from fitting equation (S10) to degradation data of various parameters from Gao et al.[19] Note that values of φ_N are multiplied by three orders of magnitude to make interpretation more manageable.

**Gao et al. (1999)**	**0V Bias**		**4V Bias**	
	$\varphi_N \times 10^3$	k	$\varphi_N \times 10^3$	k
ε_+	13	0.55	3	0.78
ε_-	16	0.57	4	0.77

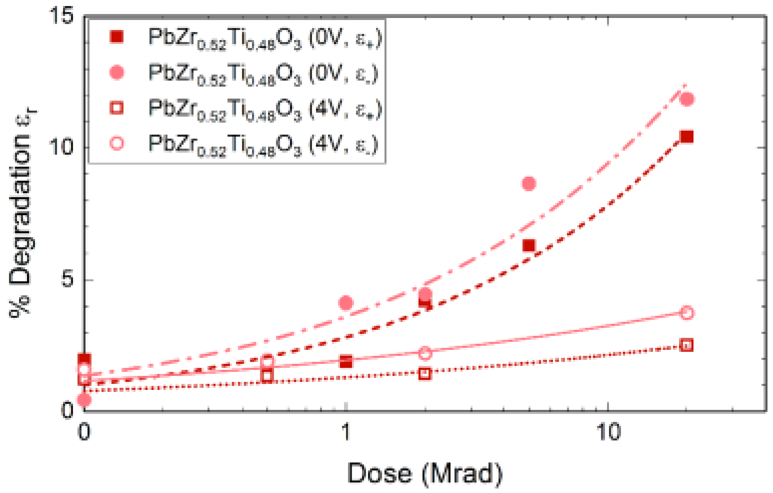

Supplementary Figure 3. Application of phenomenological model (curves) to work done by on the effects of bias conditions on gamma radiation-induced degradation of dielectric permittivity measured at the positive and negative coercive fields (ε_{r+} and ε_{r-}) of PZT thin films grown via pulsed laser deposition (PLD).[19]

Supplementary Table 3. Extracted φ_N and k parameters from fitting equation (S10) to degradation data of dielectric permittivity measurements on $PbZr_{0.52}Ti_{0.48}O_3$ and $PbTiO_3$.[17] Note that values of φ_N are multiplied by three orders of magnitude to make interpretation more manageable.

**Gao et al. (1998)**	**ε_+**		**ε_-**	
	$\varphi_N \times 10^3$	k	$\varphi_N \times 10^3$	k
$PbZr_{0.52}Ti_{0.48}O_3$	3	0.79	2	0.76
$PbTiO_3$	1	0.70	2	0.77

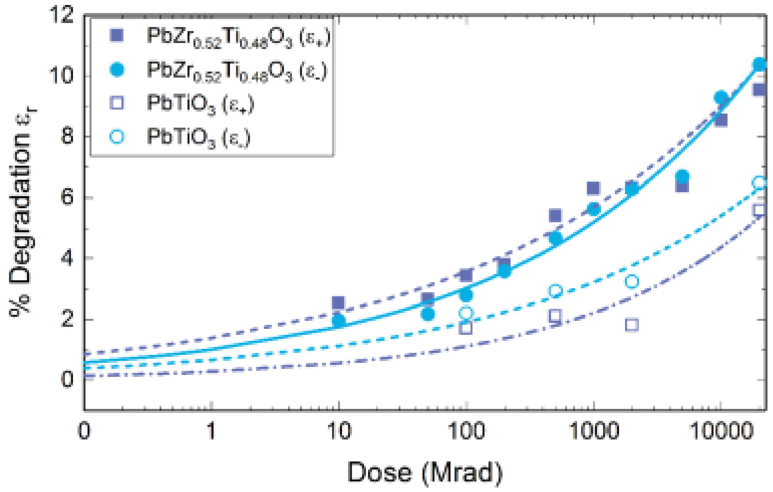

Supplementary Figure 4. Application of phenomenological model (curves) to work done on the effects of gamma irradiation on degradation of dielectric permittivity measured at the positive and negative coercive fields (ε_{r+} and ε_{r-}) in ferroelectric PZT and lead titanate thin films.[17]

Supplementary Table 4. Extracted φ_N and k parameters from fitting equation (S10) to degradation data of various parameters from Solovev et al.[18] Note that values of φ_N are multiplied by three orders of magnitude to make interpretation more manageable.

**Solovev et al.**	**ε_r**	
	$\varphi_N \times 10^3$	k
$SrTiO_3$	-1	0.95
$PbZrO_3$	5	0.79
$BaTiO_3$	6	0.80
$NaNbO_3$	6	0.82
$PbTiO_3$	14	0.75

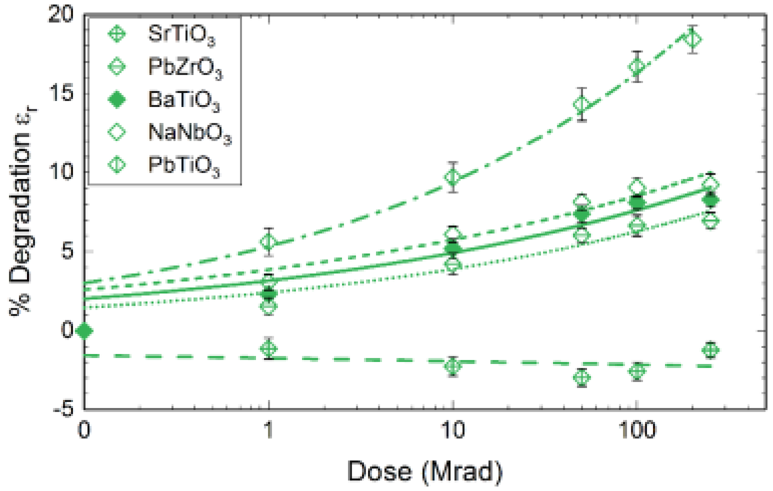

Supplementary Figure 5. Application of phenomenological model (curves) to work done on gamma irradiation of bulk ceramic ferroelectrics and corresponding changes to dielectric permittivity.[18]

Supplementary Table 5. Extracted φ_N and k parameters from fitting equation (S10) to degradation data of various parameters from Bastani et al.[12] Note that values of φ_N are multiplied by three orders of magnitude to make interpretation more manageable.

**Bastani et al.**	**X-rays**		**Protons**	
	$\varphi_N \times 10^3$	k	$\varphi_N \times 10^3$	k
ε_r (low-field)	76	0.41	26	0.57
$P_{remanent}$	79	0.57	50	0.40
ε_{init}	74	0.24	36	0.53
α	244	0.44	90	0.59
$\alpha/\varepsilon_{init}$	251	0.38	110	0.54
$d_{33,f,saturation}$	126	0.37	70	0.51

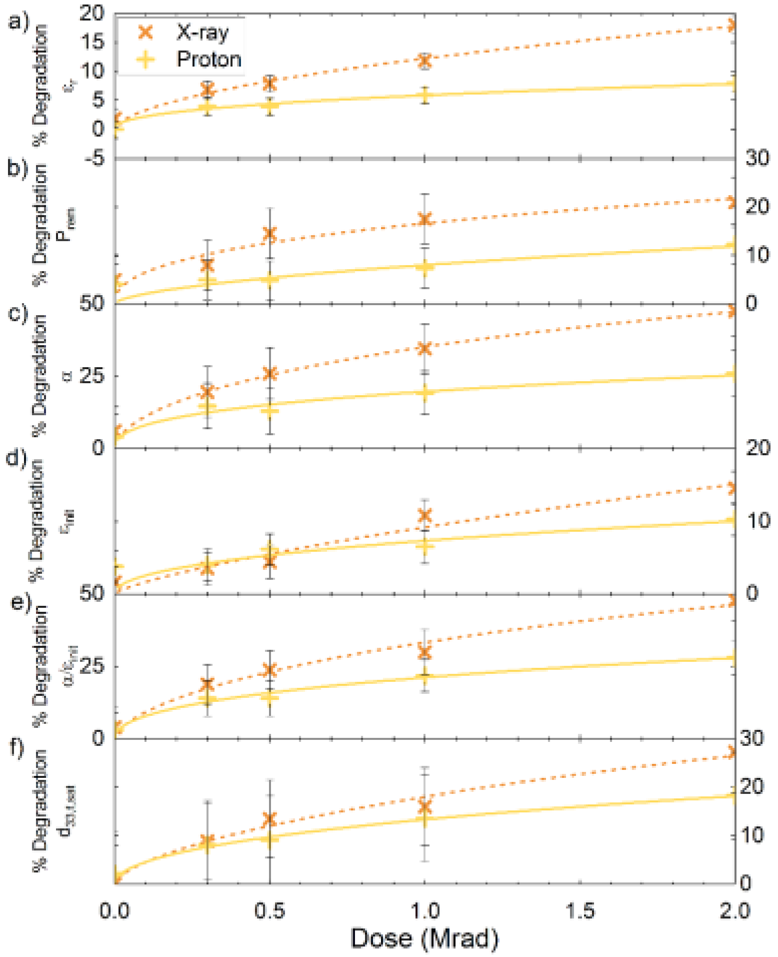

Supplementary Figure 6. Application of phenomenological model (curves) to degradation of various functional responses of PZT films subjected to X-ray and proton irradiation.[12]

Supplementary Table 6. Extracted φ_N and k parameters from fitting equation (S10) to dielectric degradation data as a function of neutron irradiation in PZT thin films by Graham et al.[14] Note that values of φ_N are multiplied by three orders of magnitude to make interpretation more manageable, and that fitting was done with neutron flux × 10^{15} cm^{-2}.

Graham et al.	**$PbZr_{0.52}Ti_{0.48}O_3$ - CSD**		**$PbZr_{0.52}Ti_{0.48}O_3$ - IMO**	
	$\varphi_N \times 10^3$	k	$\varphi_N \times 10^3$	k
ε_r (~5 kV cm^{-1})	75	0.81	47	-0.20
ε_r (~16 kV cm^{-1})	82	0.79	43	-0.36

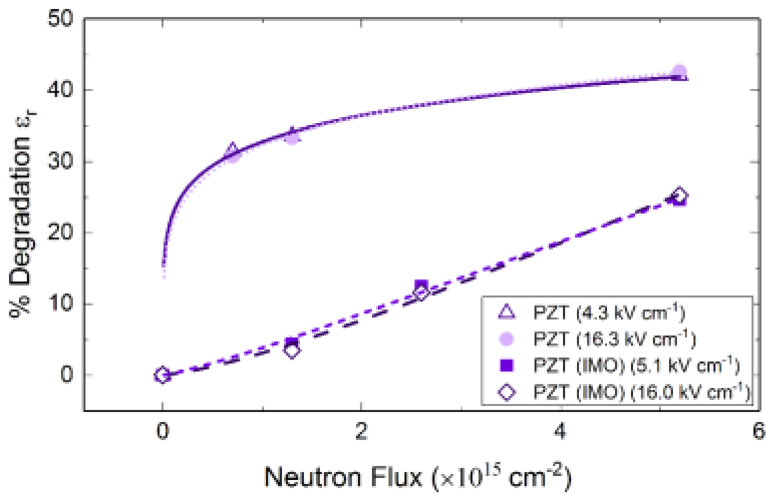

Supplementary Figure 7. Application of phenomenological model (curves) to historical data on dielectric permittivity of neutron-irradiated in PZT thin films prepared via chemical solution deposition (CSD). Inverted mixing order (IMO) methods were used on two of the films. Non-IMO films are 350 nm thick and IMO films are 320 nm thick.[14]

Supplementary Table 7. Extracted φ_N and k parameters from fitting equation (S10) to dielectric degradation data as a function of Ho_2O_3 dopant concentration in $BaTiO_3$ ceramics by Paunovic et al.[21] Note that values of φ_N are multiplied by three orders of magnitude to make interpretation more manageable.

**Paunovic et al.**	**1320K sinter**				**1380k sinter**			
	**300K measure**		**T_C measure**		**300K measure**		**T_C measure**	
	$\varphi_N \times 10^3$	k	$\varphi_N \times 10^3$	k	$\varphi_N \times 10^3$	k	$\varphi_N \times 10^3$	k
ε_+	623	0.40	963	0.17	483	0.06	1682	0.03

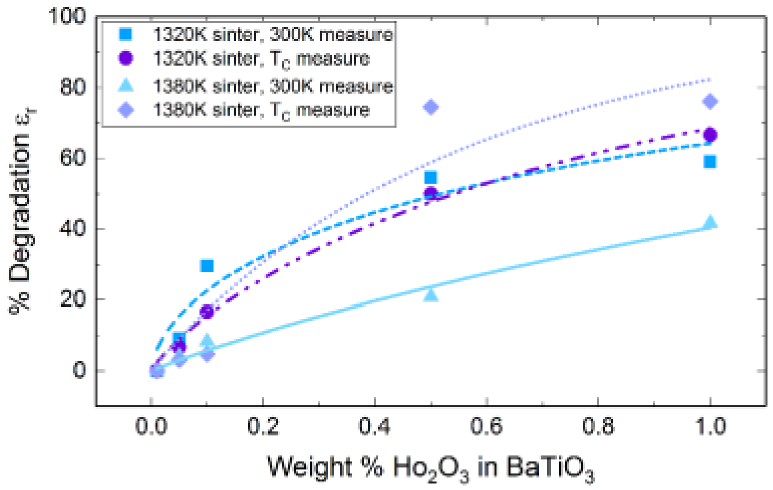

Supplementary Figure 8. Application of phenomenological model (curves) to historical data on dielectric permittivity as a function of Ho_2O_3 dopant concentration in $BaTiO_3$ ceramics. Trends are shown for various sinter temperatures and measurement temperatures, including the Curie temperature, T_C.[21]

Supplementary Table 8. Extracted φ_N and k parameters from fitting equation (S10) to dielectric degradation data as a function of Fe dopant in PZT by Weston et al.[20] Note that values of φ_N are multiplied by three orders of magnitude to make interpretation more manageable.

**Weston et al.**	**$PbZr_{0.518}Ti_{0.482}O_3$**		**$PbZr_{0.535}Ti_{0.465}O_3$**	
	$\varphi_N \times 10^3$	k	$\varphi_N \times 10^3$	k
ε_r	279	0.15	445	0.06

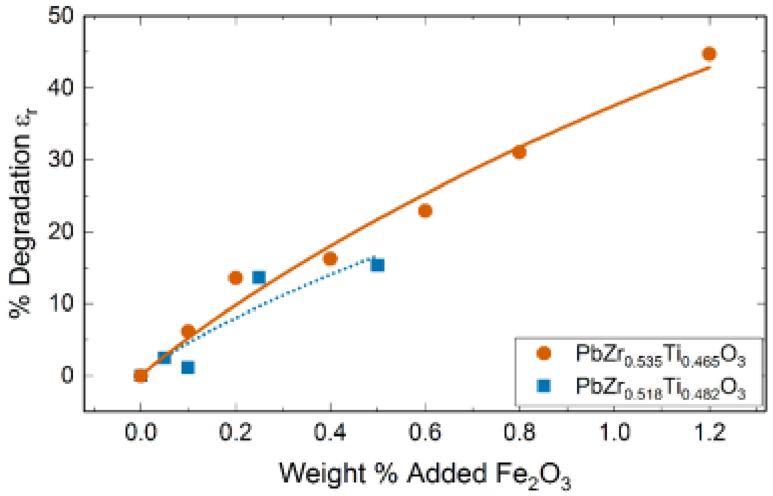

Supplementary Figure 9. Application of phenomenological model (curves) to historical data on dielectric permittivity as a function of Fe_2O_3 dopant concentration in PZT ceramics.[20]

Supplementary Table 9. Extracted φ_N and k parameters from fitting equation (S10) to degradation of conductivity in superconducting yttrium barium copper oxide (YBCO) by Clark et al.[26] Notably, the range of exposure doses for As ions is shorter than that of the O ions, but results in greater degradation and higher φ_N, suggesting that As ion bombardment is likely associated with a different degradation mechanism compared to O ions. Note that values of φ_N are multiplied by three orders of magnitude to make interpretation more manageable.

**Clark et al.**	**$Y_{0.9}Ba_{2.28}Cu_3O_x$ (500 keV O ion)**		**$Y_{0.9}Ba_{2.28}Cu_3O_x$ (1 MeV As ion)**		**$Y_{0.69}Ba_{1.89}Cu_3O_x$ (500 keV O ion)**	
	$\varphi_N \times 10^3$	k	$\varphi_N \times 10^3$	k	$\varphi_N \times 10^3$	k
Normalized Conductivity	1330	0.01	3540	0.44	1020	0.03

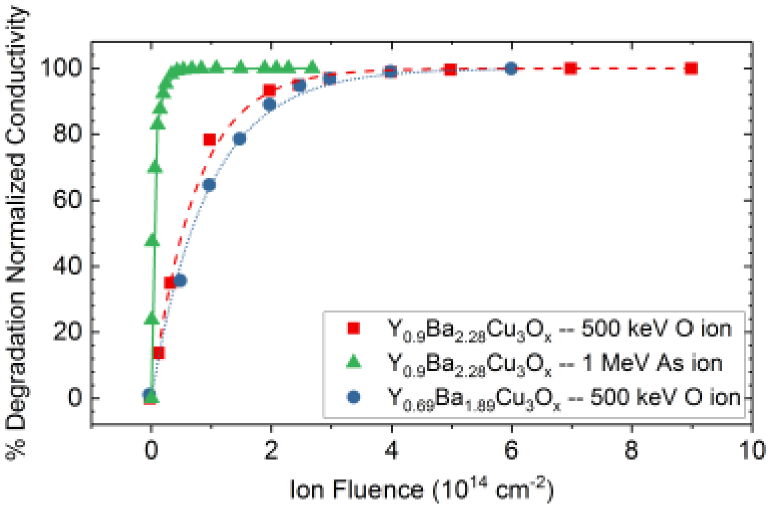

Supplementary Figure 10. Application of phenomenological model (curves) to data on irradiation of yttria barium copper oxide (YBCO) in multiple stoichiometries, irradiated with O and As ions.[26]

Supplementary Table 10. Extracted φ_N and k parameters from fitting equation (S10) to current density and polarization response degradation data as a function of He^{2+}-ion bombardment in ferroelectric thin films by Saremi et al.[27] Note that values of φ_N are multiplied by three orders of magnitude to make interpretation more manageable.

**Saremi et al.**	***Current Density***						***Polarization Properties***			
	**50 kV/cm**		**100 kV/cm**		**200 kV/cm**		**P_{rem}**		**E_{C+}**	
	$\varphi_N \times 10^3$	k	$\varphi_N \times 10^3$	k	$\varphi_N \times 10^3$	k	$\varphi_N \times 10^3$	k	$\varphi_N \times 10^3$	k
ε_+	611	0.004	547	0.13	410	0.00	64	0.55	-25	0.89

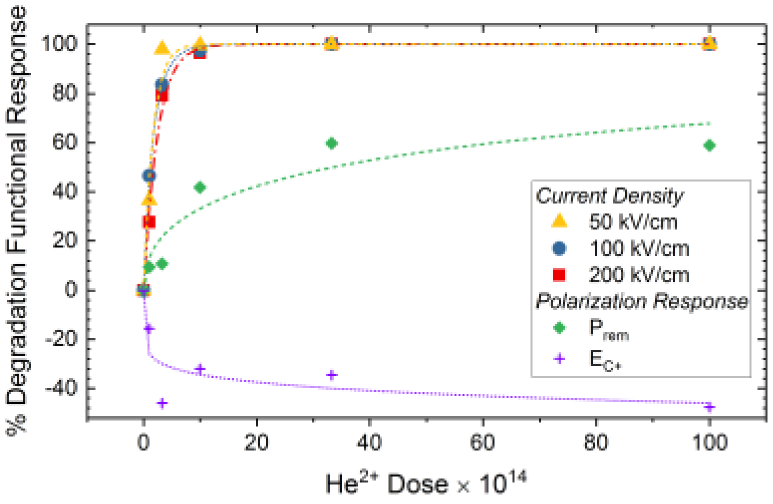

Supplementary Figure 11. Application of phenomenological model (curves) to data on He^{2+}-ion bombardment of epitaxial ferroelectric thin films.[27]

Supplementary Table 11. Extracted φ_N and k parameters from fitting equation (S10) to degradation of total conductivity of In dopant yttrium barium cerate, compiled by Medvedev.[28] Note that values of φ_N are multiplied by three orders of magnitude to make interpretation more manageable.

**Medvedev**	**σ (550 °C)**		**σ (750 °C)**	
	$\varphi_N \times 10^3$	k	$\varphi_N \times 10^3$	k
Wet Air	7200	0.05	3917	0.28
Dry Air	3590	0.40	4538	0.25
Wet H_2	11682	-0.13	5424	0.22
Wet N_2	13390	-0.13	6313	0.15

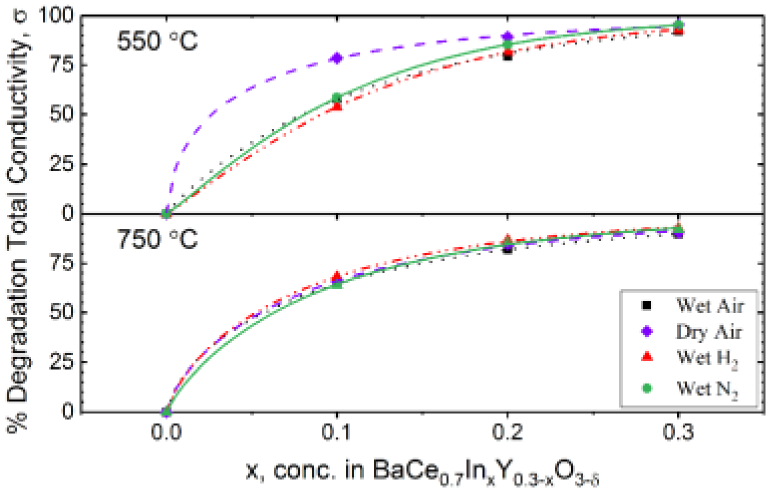

Supplementary Figure 12. Application of phenomenological model (curves) to In-doping of yttrium barium cerate at multiple atmospheric conditions, for use in solid oxide fuel cell (SOFC) applications.[28]

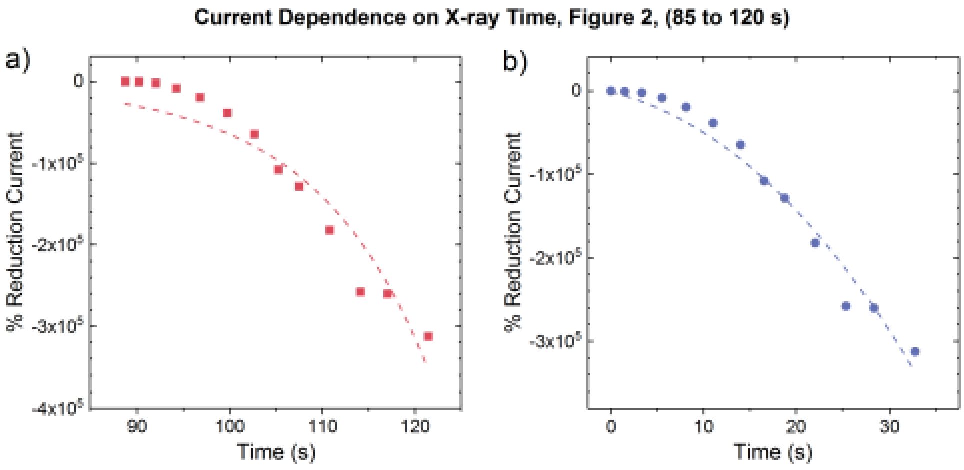

Supplementary Figure 13. Application of phenomenological model (curves) to the effects of X-ray synchrotron experiments on current in Pt/TiO_2/Pt memory cells.[29] Data is extracted Figure 2 of the work by Chang *et al.*, from

approximately 85 to 120 seconds, due to the continuous set of data at that location. Data has been normalized to the first point of the selected set, and the percent change from that point as the baseline plotted. The model fits well in (a), but does encounter some difficulty due to the starting point at 85 s and resulting discontinuity from 0 to 85 s. By shifting the initial point to 0 s (b), the model provides a better fit. We note that the model requires fitting the degradation/reduction of a property as a positive value (as explained in the manuscript), thus the negative trends here represent an increase to the electric current.

III. Crystallographic Phase Analysis

Crystallographic phase analysis was performed to confirm the texture of PZT samples prepared via 2-methoxyethanol-based (2-MOE) and acetic acid-based inverted mixing order (IMO) chemical solution deposition (CSD) processes (see Experimental Section). Supplementary Figure 14 shows baseline X-ray diffraction (XRD) phase analysis of the films deposited using 2-MOE-based and IMO-prepared solutions, and the resulting columnar and equiaxed grain morphologies, respectively.

Noteworthy are the large 100- and 200-textures in the columnar sample, indicating highly-textured samples; and the relatively large 101-peak in the equiaxed sample compared to the 100- and 200-peaks, indicating their more equiaxial nature and random orientation.

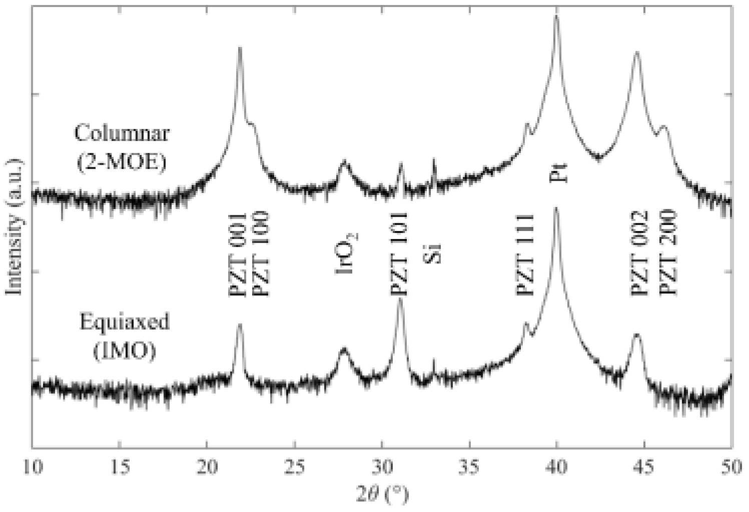

Supplementary Figure 14. X-ray diffraction crystallographic phase analysis comparing representative samples with columnar (top) and equiaxed (bottom) grain structures prepared using 2-MOE and IMO PZT solutions, respectively. Note the large relative intensity of the 100-peak for samples with columnar grains while the opposite behavior for the 101-peak is found for samples with equiaxed structure.

IV. TEM/TKD and Statistical Analysis

Cross-sectional transmission electron microscopy (TEM) and transmission Kikuchi diffraction (TKD) were performed at North Carolina State University (NC State) to observe domain and grain structure in the samples (Figure 3b, 3e). The regions of mottled contrast, primarily visible in the sample with columnar grains (Figure 3b), indicate potential nanodomains or striated 90° domain walls.[30,31]

TKD and TEM images were analyzed to calculate statistical values of several grain size characteristics, including mean in-plane grain size, mean out-of-plane grain height, and mean size of multi-grain regions of similar dimension (Supplementary Table 12). These statistics aid in analysis of samples with columnar vs. equiaxed grains.

Supplementary Table 12. Statistical size measurements for samples with columnar and equiaxed grain structures, based on measurements of mean grain height and width (in-plane size). Columnar grains are assumed to be cylindrical, with the axis normal to the substrate, while equiaxed grains are assumed to be ellipsoidal, with the longest axis normal to the substrate and the remaining two axes equal to the in-plane grain size.

Grain structure	Mean grain height (nm)	Mean in-plane grain size (nm)	Mean grain surface area (cm^2)	Mean grain volume (cm^3)	Surface area per volume	Number of electron-hole pairs (ehp) per grain·Mrad
**Columnar (cylindrical)**	391 ± 220	66 ± 27	8.8×10^{-10}	1.3×10^{-15}	6.8×10^{5}	1.2×10^{5}
**Equiaxed (ellipsoidal)**	215 ± 109	177 ± 160	11.3×10^{-10}	3.6×10^{-15}	3.1×10^{5}	3.4×10^{5}

V. Functional Response Characterization

Dielectric, polarization, and electromechanical responses of the samples were fully characterized at Georgia Institute of Technology both before and after irradiation, including measurements of low-field permittivity and DC electric field-dependent electromechanical response, followed by irradiation and repetition of experiments (see Experimental Section). A summary of these measurements for samples with both columnar and equiaxed grain structures as a function of radiation dose is shown in Supplementary Table 13. A 600 second poling step at 10 V, approximately five times the coercive voltage, V_c, was performed directly before the electromechanical measurements in both pre- and post-irradiation measurement sets in order to eliminate anisotropic polarization contributions to the electromechanical response. All measurements were performed on the same sample/electrode both before and after irradiation in order to monitor precise changes in response behavior. Low-field dielectric permittivity (ε_r) measurements were conducted at 100 mV and 1 kHz using an Agilent 4284A precision LCR meter. Measurements of the converse, effective longitudinal piezoelectric response ($d_{33,f}$) were performed on an aixACCT double beam laser interferometer (DBLI) measurement system up to 300 kV cm^{-1} DC bias with an overlapping AC signal $V_{AC} \approx 0.5\ V_c$. All measurements reported are subject to experimental error up to 3-5%, due to sample variability. From the data in Supplementary Table 13, degradation trends were extracted by calculating the percent change in response from the virgin control sample. This data has been tabulated in Supplementary Table 14.

Supplementary Table 13. Measured dielectric, polarization, and electromechanical responses at increasing radiation doses, for PZT thin films with columnar and equiaxed grain structures. Percent change is measured from 0 to 10 Mrad radiation exposure. Uncertainties expressed represent standard error from the mean to one significant figure. Measurement values are reported to the same decimal place as uncertainty for said measurement.[32]

	Dose (Mrad)	Virgin	0.2	0.5	1.0	2.0	5.0	10.0
ε_r	Col	1222 ± 6	1297 ± 10	1229 ± 4	1208 ± 4	1188 ± 3	1127 ± 4	1083 ± 2
	Eqx	1357 ± 4	1323 ± 2	1215 ± 6	1284 ± 4	1284 ± 5	1262 ± 6	1175 ± 2
$d_{33,f,remanent}$ (pm V^{-1})	Col	34 ± 4	28 ± 1	25 ± 1	22 ± 1	23 ± 2	24 ± 6	7 ± 2
	Eqx	43 ± 3	48 ± 6	25 ± 3	33 ± 3	30 ± 4	30 ± 2	19 ± 5
$d_{33,f,saturation}$ (pm V^{-1})	Col	79 ± 2	76 ± 1	75 ± 0	76 ± 3	78 ± 1	76 ± 1	77 ± 2
	Eqx	68 ± 3	64 ± 1	63 ± 1	64 ± 1	59 ± 2	64 ± 1	64 ± 6

Supplementary Table 14. Mean percent change in measured dielectric, polarization, and electromechanical responses as a function of radiation dose, relative to virgin control sample, for PZT thin films with columnar and equiaxed grain structures. Negative numbers represent a degradation of response. All measurements are subject to a 3 to 5% standard measurement error.

	Dose (Mrad)	Virgin	0.2	0.5	1.0	2.0	5.0	10.0
	% Change	%	%	%	%	%	%	%
ε_r	Col	-1	4	0	-2	-4	-9	-13
	Eqx	-3	-11	-11	-15	-11	-13	-22
$d_{33,f,remanent}$	Col	-31	-26	-42	-44	-46	-45	-83
	Eqx	9	-35	-48	-43	-35	-44	-70
$d_{33,f,saturation}$	Col	0	0	-2	-2	-2	-3	-5
	Eqx	1	-7	-7	1	0	3	0

VI. Electron-Hole Pairs Generated by Gamma Irradiation

Exposure to gamma radiation results in the formation of electron-hole pairs (ehp) in ferroelectric PZT. Work by Leray et al., showed that radiation dose in PZT is 1.23 times that of the dose felt in Si, i.e. 1 Mrad(Si) = 1.23 Mrad (PZT).[33] Using a mean density of PZT as 7.6 g cm^{-3} and 100 rad = 1 Gy = 1 J kG^{-1} we can calculate the total energy deposited:

$$Energy\ Deposited = 5.84 \times 10^{20}\ eV\ cm^{-3}\ Mrad(Si)^{-1}$$

Using 6.25 eV as the mean energy per ehp, we arrive at

$$Number\ of\ ehp = 9.34 \times 10^{10}\ ehp\ cm^{-3}\ Mrad(Si)^{-1}$$

We can apply this result to calculate the number of ehp per grain. Assuming that columnar grains are roughly cylindrical (with axis normal to substrate), and equiaxed grains are ellipsoidal (with a = grain height and b = c = in-plane grain size), we can calculate the number of ehp per grain from data extracted from Figure 3. Supplementary Table 12 shows these calculations and resulting number of ehp per grain—the results indicate that over twice as many ehp are generated per equiaxed grain compared to columnar grains (3.5×10^5 ehp/equiaxed vs. 1.4×10^5 ehp/columnar).

References

1 Fowler, J. F. X-Ray induced conductivity in insulating materials. *Proceedings of the Royal Society of London Series A-Mathematical and Physical Sciences* **236**, 464-480, doi:DOI 10.1098/rspa.1956.0149 (1956).

2 Oen, O. S. & Holmes, D. K. Cross sections for atomic displacements in solids by gamma-rays. *J. Appl. Phys.* **30**, 1289-1295, doi:Doi 10.1063/1.1735307 (1959).

3 Sickafus, K. *et al.* Radiation-induced amorphization resistance and radiation tolerance in structurally related oxides. *Nat. Mater.* **6**, 217-223, doi:10.1038/nmat1842 (2007).

4 Glower, D. D., Hester, D. L. & Warnke, D. F. Effects of radiation-induced damage centers in lead zirconate titanate ceramics. *J. Am. Cer. Soc.* **48**, 417-&, doi: 10.1111/j.1151-2916.1965.tb14780.x (1965).

5 Gao, J. *et al.* Characteristics of Au/$PbZr_{0.52}Ti_{0.48}O_3$/$YBa_2Cu_3O_{7-\delta}$ capacitors fabricated on $LaAlO_3$ and Y_2O_3-stabilized ZrO_2 substrates during irradiation. *Phil. Mag. Part B* **79**, 829-838, doi:10.1080/13642819908214843 (1999).

6 Gao, J. *et al.* Total dose radiation effects of Au/$PbZr_{0.52}Ti_{0.48}O_3$/$YBa_2Cu_3O_{7-\delta}$ capacitors. *Thin Solid Films* **340**, 132-136, doi:10.1016/S0040-6090(98)01368-6 (1999).

7 Yang, S. A. *et al.* Gamma-ray irradiation effects on electrical properties of ferroelectric $PbTiO_3$ and $Pb(Zr_{0.52}Ti_{0.48})O_3$ thin films. *Thin Solid Films* **562**, 185-189, doi:http://dx.doi.org/10.1016/j.tsf.2014.04.038 (2014).

8 Baturin, I. *et al.* Influence of irradiation on the switching behavior in PZT thin films. *Mater. Sci. Eng.: B* **120**, 141-145, doi:http://dx.doi.org/10.1016/j.mseb.2005.02.024 (2005).

9 Menou, N. *et al.* Degradation and recovery of polarization under synchrotron X-rays in $SrBi_2Ta_2O_9$ ferroelectric capacitors. *J. Appl. Phys.* **97**, 044106, doi:doi:http://dx.doi.org/10.1063/1.1851598 (2005).

10 Scott, J. F., Araujo, C. A., Meadows, H. B., McMillan, L. D. & Shawabkeh, A. Radiation effects on ferroelectric thin film memories: Retention failure mechanisms. *J. Appl. Phys.* **66**, 1444-1453, doi:doi:http://dx.doi.org/10.1063/1.344419 (1989).

11 Brewer, S. *et al.* Effect of top electrode material on radiation-induced degradation of ferroelectric thin film structures. *J. Appl. Phys.* **120**, doi:10.1063/1.4955424 (2016).

12 Bastani, Y. *et al.* Effects of high energy x ray and proton irradiation on lead zirconate titanate thin films' dielectric and piezoelectric response. *Applied Physics Letters* **102**, doi: 10.1063/1.4805045 (2013).

13 Proie, R. M. *et al.* Total ionizing dose effects in piezoelectric MEMS relays. *IEEE Trans. Nucl. Sci.* **60**, 4505-4511, doi:10.1109/Tns.2013.2282261 (2013).

14 Graham, J. T. *et al.* Neutron irradiation effects on domain wall mobility and reversibility in lead zirconate titanate thin films. *J. Appl. Phys.* **113**, doi:10.1063/1.4795869 (2013).

15 Henriques, A. *et al.* Crystallographic changes in lead zirconate titanate due to neutron irradiation. *AIP Adv.* **4**, doi:Artn 117125

10.1063/1.4902179 (2014).
16 Zhang, G. *et al.* Dose rate dependence of electrical characteristics of lead zirconate titanate capacitors. *Jpn. J. Appl. Phys. 1* **42**, 6491-6495, doi:10.1143/JJAP.42.6491 (2003).
17 Gao, J., Zheng, L., Zeng, J. & Lin, C. Gamma-ray total dose radiation effects of Pt/$PbZr_{0.52}Ti_{0.48}$/Pt and Au/$PbTiO_3$/$YBa_2Cu_3O_{7-\delta}$ ferroelectric capacitors. *Jpn. J. Appl. Phys. 1* **37**, 5126-5127, doi:10.1143/JJAP.37.5126 (1998).
18 Solovev, S. P., Zakurkin, V. V., Kuzmin, I. I. & Dudarev, V. J. X-ray and dielectric studies of irradiated perovskite type compounds. *J. Phys. France* **33**, 443–&, doi:10.1051/jphys:01972003304044300 (1972).
19 Gao, J. *et al.* Total dose radiation effects of Pt/PZT/Pt ferroelectric capacitors fabricated by PLD method. *Semicond. Sci. Tech.* **14**, 836-839, doi:10.1088/0268-1242/14/9/315 (1999).
20 Weston, T. B., Webster, A. H. & McNamara, V. M. Lead zirconate-lead titanate piezoelectric ceramics with iron oxide additions. *J. Am. Cer. Soc.* **52**, 253-&, doi:10.1111/j.1151-2916.1969.tb09178.x (1969).
21 Paunovic, V., Mitic, V., Miljkovic, M., Pavlovic, V. & Zivkovic, L. Ho_2O_3 additive effects on $BaTiO_3$ ceramics microstructure and dielectric properties. *Sci. Sinter.* **44**, 223-233, doi:10.2298/SOS1202223P (2012).
22 Berger, M. J., Coursey, J. S., Zucker, M. A. & Chang, J. in *NISTIR 4999. NIST Physical Measurement Laboratory* (1998).
23 Ziegler, J. F. (http://www.srim.org/, 2017).
24 Berger, M. J. *NIST Physical Measurement Laboratory* (1998).
25 Oldham, T. & McLean, F. Total ionizing dose effects in MOS oxides and devices. *IEEE Trans. Nucl. Sci.* **50**, 483-499, doi:10.1109/TNS.2003.812927 (2003).
26 Clark, G., Marwick, A., Koch, R. & Laibowitz, R. Effects of radiation-damage in ion-implanted thin films of metal-oxide superconductors. *Appl. Phys. Lett.* **51**, 139-141, doi:10.1063/1.98594 (1987).
27 Saremi, S. *et al.* Enhanced electrical resistivity and properties via ion bombardment of ferroelectric thin films. *Adv. Mater.* (2016).
28 Medvedev, D., Lyagaeva, J., Gorbova, E., Demin, A. & Tsiakaras, P. Advanced materials for SOFC application: Strategies for the development of highly conductive and stable solid oxide proton electrolytes. *Prog. Mater. Sci.* **75**, 38-79, doi:10.1016/j.pmatsci.2015.08.001 (2016).
29 Chang, S. *et al.* X-ray irradiation induced reversible resistance change in Pt/TiO_2/Pt cells. *ACS Nano* **8**, 1584-1589, doi:10.1021/nn405867p (2014).
30 Theissmann, R. *et al.* Nanodomains in morphotropic lead zirconate titanate ceramics: On the origin of the strong piezoelectric effect. *J. Appl. Phys.* **102**, doi:10.1063/1.2753569 (2007).
31 Lucuta, P. G., Teodorescu, V. & Vasiliu, F. SEM, SAED, and TEM investigations of domain structure in PZT ceramics at morphotropic phase boundary. *Appl. Phys. A-Mater. Sci. Proc.* **37**, 237-242, doi:10.1007/BF00614823 (1985).
32 Taylor, J. R. *An introduction to error analysis : the study of uncertainties in physical measurements*. 2nd edn, (University Science Books, 1997).
33 Leray, J. *et al.* Radiation effects in thin-film ferroelectric PZT for non-volatile memory applications in microelectronics. *J. Phys. III* **7**, 1227-1243 (1997).
